# Using newborn screening analytes to identify cases of neonatal sepsis

**DOI:** 10.1038/s41598-017-18371-1

**Published:** 2017-12-21

**Authors:** Deshayne B. Fell, Steven Hawken, Coralie A. Wong, Lindsay A. Wilson, Malia S. Q. Murphy, Pranesh Chakraborty, Thierry Lacaze-Masmonteil, Beth K. Potter, Kumanan Wilson

**Affiliations:** 1 School of Epidemiology and Public Health, University of Ottawa, Ottawa Ontario, Canada; 2 Children’s Hospital of Eastern Ontario Research Institute, Ottawa Ontario, Canada; 3 Institute for Clinical Evaluative Sciences (ICES), University of Ottawa, Ottawa Ontario, Canada; 4 Clinical Epidemiology Program, Ottawa Hospital Research Institute, Ottawa Ontario, Canada; 5 Department of Pediatrics, University of Ottawa, Ottawa Ontario, Canada; 6 Newborn Screening Ontario (NSO), Children’s Hospital of Eastern Ontario, Ottawa Ontario, Canada; 7 Department of Pediatrics, University of Calgary, Calgary Alberta, Canada; 8 Department of Medicine, University of Ottawa, Ottawa Ontario, Canada

## Abstract

Neonatal sepsis is associated with high mortality and morbidity, yet challenges with available diagnostic approaches can lead to delays in therapy. Our study assessed whether newborn screening analytes could be utilized to identify associations with neonatal sepsis. We linked a newborn screening registry with health databases to identify cases of sepsis among infants born in Ontario from 2010–2015. Correlations between sepsis and screening analytes were examined within three gestational age groups (early preterm: <34 weeks; late preterm: 34–36 weeks; term: ≥37 weeks), using multivariable logistic regression models. We started with a model containing only clinical factors, then added groups of screening analytes. Among 793,128 infants, 4,794 were diagnosed with sepsis during the neonatal period. Clinical variables alone or in combination with hemoglobin values were not strongly predictive of neonatal sepsis among infants born at term or late preterm. However, model fit improved considerably after adding markers of thyroid and adrenal function, acyl-carnitines, and amino acids. Among infants born at early preterm gestation, neither clinical variables alone nor models incorporating screening analytes adequately predicted neonatal sepsis. The combination of clinical variables and newborn screening analytes may have utility in identifying term or late preterm infants at risk for neonatal sepsis.

## Introduction

Neonatal sepsis, broadly defined as a systemic infection of predominantly bacterial origin in neonates^1–3^, is a leading cause of infant mortality and morbidity around the world^1,4–10^. Even in high-resource countries where the incidence of confirmed neonatal sepsis is relatively low (1–5 cases per 1,000 live births^3,5,9,11,12^) and all such infants would receive treatment, approximately 40% will either die or suffer major developmental disabilities^13^. Although reported incidence rates of neonatal sepsis from low-resource countries are generally higher^14–16^, cases are likely underascertained owing to many factors, including a lack of access to care, poor quality care, and a lack of adequate laboratory services^16,17^. In one review of community-based studies from low- and middle-income countries, incidence rates ranged from 49 to 170 cases per 1,000 live births across 11 studies^16^.

The successful clinical management of neonatal sepsis is predicated on early detection and prompt initiation of antibiotic therapy; however, this is difficult to achieve in practice owing to the non-specific signs and symptoms of sepsis, low sensitivity of the gold standard blood culture test (particularly following intrapartum antibiotic prophylaxis), and delayed availability of culture results (approximately 48–72 hours after blood collection)^7–9,12,18–21^. To prevent negative health outcomes in the face of these diagnostic challenges, antibiotic therapy is generally initiated in all clinically-suspected cases of neonatal sepsis^22^. However, concerns are being increasingly raised about this recommended approach^22,23^, as it contributes to unnecessary or prolonged antibiotic therapy, antimicrobial resistance, and heightened risk of nosocomial infection due to increased length of hospital stay while the infant is monitored^10,12,24^.

Given current challenges with diagnosis, and the high mortality and morbidity associated with neonatal sepsis, particularly in low-income countries, there is a need to develop novel approaches for identifying neonates at greatest risk. Numerous biomarkers have been studied to assess their ability to identify neonatal sepsis, but thus far no individual biomarkers have demonstrated adequate sensitivity and specificity^3,8,12,23^ and many have important limitations that preclude their utility for neonatal sepsis diagnosis^25^. High-dimensional biology, including the study of the metabolome, is increasingly being used to help provide insights on complex biological processes and disorders^26^. Newborn screening measures a diverse panel of analytes, including amino acids, acyl-carnitines, hemoglobin variants, and enzymatic and endocrine markers to identify infants with rare, treatable conditions. We hypothesized that newborn screening analytes — either individually or in combination^12,25,27^ — could be utilized beyond their traditional application to identify neonates at high risk for sepsis. For example, differences in these analytes could reflect underlying susceptibility to neonatal sepsis through suboptimal metabolic responses to the physiologic stress associated with infection. Alternatively, any differences could also represent the sequelae of early subclinical infection as a manifestation of associated catabolic stress. Our primary objective, therefore, was to assess whether there was any association between newborn screening analyte profiles and neonatal sepsis in a large, population-based cohort of infants.

## Results

### Characteristics of study population

**Figure 1:**
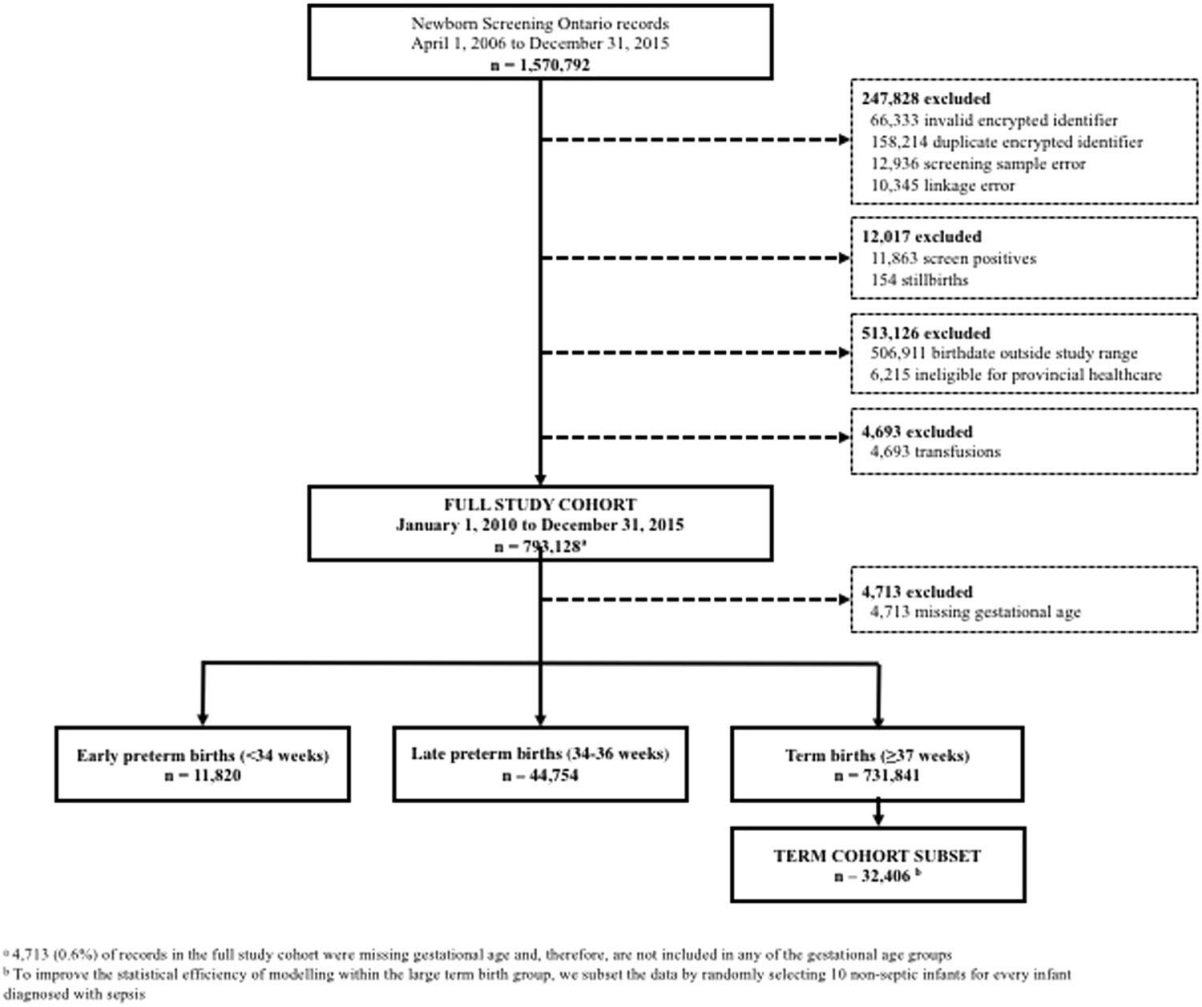
Study flow diagram.

Overall, there were 793,128 infants in the full study cohort following exclusions (Fig. 1), 4,794 of whom received a diagnosis of sepsis during the neonatal period (6.04 per 1,000 screened infants; Table 1). Rates of sepsis were highest among infants born at early preterm gestation (86.6 per 1,000) and decreased with increasing gestational age at birth. Almost 40% of the total number of cases of neonatal sepsis occurred among infants born prior to 37 weeks’ gestation (Table 1). Rates of sepsis were also higher among neonates with a birth weight under 2,500 grams and among those born from a multifetal gestation. The timing of sample collection was later among infants diagnosed with sepsis during the neonatal period (median of 44 hours) compared to the total population (median of 28 hours). After excluding 4,713 infants with missing gestational age information, there were 731,841 infants born at term gestation (92.8%), 44,754 late preterm infants (5.7%), and 11,820 early preterm infants (1.5%). Corresponding rates of neonatal sepsis per 1,000 screened infants within each of these gestational age groups were: 4.03, 18.0, and 86.6, respectively. The distribution of baseline characteristics in the term cohort subset was similar to the full group of term births (Table 2).

**Table 1:** Characteristics of the study population and frequency of neonatal sepsis.

Characteristics	Full study population n = 793,128		Neonatal sepsis n = 4,794		
	n	%	n	%	Rate per 1,000 screened infants
All infants	793,128	100	4,794	100	6.0
Infant sex					
Female	387,221	48.8	2,084	43.5	5.4
Male	405,901	51.2	2,710	56.6	6.7
Unknown	6	0.0	0	0.0	—
Gestational age (completed weeks)					
Mean ± SD	38.86 ± 1.72^a^		36.63 ± 3.92^b^		
<34	11,820	1.5	1,024	21.4	86.6
34–36	44,754	5.6	805	16.8	18.0
≥37	731,841	92.3	2,946	61.5	4.0
Missing	4,713	0.6	19	0.4	4.0
Birth weight (grams)					
Mean ± SD	3,355.01 ± 550.98^c^		2,882.14 ± 947.01^d^		
<2,500	46,131	5.8	1,558	32.5	33.8
≥2,500	745,530	94.0	3,225	67.3	4.3
Missing	1,467	0.2	11	0.2	7.5
Multiple birth					
No	765,350	96.5	4,290	89.5	5.6
Yes	27,778	3.5	504	10.5	18.1
Age at blood spot collection (hours)					
Median (IQR)	28.45 (24.67–42.72)		43.81 (25.73–72.77)		
≤72	739,436	93.2	3,565	74.36	4.8
>72	53,682	6.8	1,229	25.6	22.9
Missing	10	0.0	0	0.0	—
Any total parenteral nutrition^e^					
No	785,907	99.1	4,163	86.8	5.3
Yes	6,172	0.8	629	13.1	101.9
Missing	1,049	0.1	^f^	—	—
Neonatal death^g^					
No	792,879	100.0	4,750	99.1	60.
Yes	249	0.0	44	0.9	176.7

IQR: inter-quartile range; SD: standard deviation.

^a^Mean (SD) within gestational age groups in full study population: <34 weeks: 31.2 (1.97); 34–36 weeks: 35.4 (0.76); ≥37 weeks: 39.2 (1.15).

^b^Mean (SD) within gestational age groups in infants diagnosed with neonatal sepsis: <34 weeks: 30.3 (2.24); 34–36 weeks: 35.0 (0.84); ≥37 weeks: 39.3 (1.23).

^c^Mean (SD) within birth weight groups in full study population: <2,500 grams: 2,099 (278); ≥2,500 grams: 3,433 (458).

^d^Mean (SD) within birth weight groups in infants diagnosed with neonatal sepsis: <2,500 grams: 1,731 (475); ≥2,500 grams: 3,438 (522).

^e^Total parenteral nutrition alone or in combination with other infant feeding method.

^f^<6 infants had missing information. The number has been combined with the ‘no’ category.

^g^Rate of neonatal death: without a diagnosis of neonatal sepsis: 0.3 per 1,000; with a diagnosis of neonatal sepsis: 9.2 per 1,000.

**Table 2:** Characteristics of the study population by gestational age group.

Characteristics	Early preterm births (<34 weeks’ gestation) n = 11,820		Late preterm births (34–36 weeks’ gestation) n = 44,754		Term births (≥37 weeks’ gestation)			
					All n = 731,841		Term cohort subset n = 32,406	
	n	%	n	%	n	%	n	%
All infants	11,820	100	44,754	100	731,841	100	32,406	100
Neonatal sepsis	1,024	86.6 ^a^	805	18.0^a^	2,946	4.0^a^	2,946	—^b^
Infant sex								
Female	5,386	45.6	20,666	46.2	353,838	49.0	15,694	48.4
Male	6,434	54.4	24,088	53.8	372,997	51.0	16,712	51.6
Unknown	0	0.0	0	0.0	6	0.0	0	0.0
Birth weight (grams)								
Mean ± SD	1,730 ± 510.12		2,602.32 ± 482.02		3,426.38 ± 476.52		3,428.85 ± 485.75	
<2,500	11,106	94.0	18,940	42.3	15,972	2.2	733	2.4
≥2,500	704	6.0	25,779	57.6	714,784	97.7	31,593	97.5
Missing	10	0.1	35	0.1	1,085	0.1	40	0.1
Multiple birth								
No	8,058	68.2	34,318	76.7	718,308	98.2	31,792	98.1
Yes	3,762	31.8	10,436	23.3	13,533	1.8	614	1.9
Age at blood spot collection (hours)								
Median (IQR)	73.03 (36.17–118.38)		38.57 (26.13–60.42)		27.87 (24.63–40.92)		28.15 (24.65–42.10)	
≤72	5,828	49.3	36,319	81.2	693,981	94.8	30,561	90.65
>72	5,991	50.7	8,435	18.8	37,851	5.2	1,845	5.7
Missing	^c^	—	0	0.0	9	0.0	^c^	—
Any total parenteral nutrition^d^								
No	8,214	69.5	43,438	97.1	729,557	99.7	32,227	99.4
Yes	3,597	30.4	1,259	2.8	1,301	0.2	135	0.4
Missing	9	0.1	57	0.1	983	0.1	44	0.1
Neonatal death								
No	11,751	99.4	44,725	99.9	731,692	100	32,391	100.0
Yes	69	0.6	29	0.1	149	0.0	15	0.0

IQR: inter-quartile range; SD: standard deviation.

^a^Rate per 1,000 infants.

^b^Rate not reported for the term cohort subset, as the denominator size was fixed at a ratio of 1:10 for infants with and without sepsis, respectively.

^c^<6 infants had missing information. The number has been combined with the ‘>72 hours’ category.

^d^Total parenteral nutrition alone or in combination with other infant feeding method.

### Model performance by gestational age

#### Term subset (≥37 weeks)

The results from the predictive modelling are summarized in Table 3. In the term birth subset of 32,406 infants, the addition of the relative fetal-to-adult Hb level in Model 2 to the baseline model containing only clinical characteristics (Model 1) did not improve the fit of the model, as measured by the c-statistic. However, the fit improved substantially (adjusted c-statistic increased from 0.577 in Model 1 to 0.704 in Model 3) when select non-mass spectrometry derived newborn screening analytes (17-OHP and TSH) were added, and improved further still (adjusted c-statistic of 0.848) when remaining analytes were added in the final model (Model 4). The addition of screening analytes in Models 3 and 4 also significantly improved the predicted probabilities between events and non-events compared with the preceding model (IDI values shown in Table 3). Higher levels of 17-OHP were associated with an increase in the likelihood of neonatal sepsis while higher TSH level was associated with a decreased likelihood even after other screening analytes were added in the final model.

**Table 3:** Model performance comparing baseline clinical model (Model 1) and clinical model plus newborn screening analytes for prediction of neonatal sepsis.

Model		c-statistic^a,b^	c-statistic adjusted^a,b^	Optimism correction^a,b^	AIC (lower is better)	IDI^c^ (95% CI)	NRI^d^ (95% CI)
**Term births (≥37 weeks’ gestation)**							
**Model 1** ^**e**^	Infant sex, gestational age, birth weight, plurality, and TPN	0.579	0.577	0.002	19372	—	—
**Model 2**	Model 1 variables + relative fetal-to-adult Hb level	0.580	0.577	0.003	19372	0.000075 (0, 0.0002)	0.010446 (−0.0271, 0.048)
**Model 3**	Model 2 variables + 17 − OHP + TSH	0.705	0.704	0.001	17977	0.063638 (0.0584, 0.0688)	0.55832 (0.521, 0.5957)
**Model 4** ^**f**^	Model 3 variables + restricted cubic spline terms for the top five ranked analytes/analyte ratios + remaining analytes/analyte ratios until maximum number of parameters was reached (maximum number of parameters: 294)	0.848	0.848^g^	—^g^	13788	0.21417 (0.2046, 0.2238)	0.97868 (0.9443, 1.013)
**Top 10 analytes/analyte ratios** ^h^		Tyrosine; Tyrosine:Relative fetal-to-adult Hb level; Malonylcarnitine:Relative fetal-to-adult Hb level; Tetradecenoyl carnitine:Relative fetal-to-adult Hb level; Dodecenoylcarnitine:Phenylalanine; Tetradecenoyl carnitine; Malonylcarnitine; Glutarylcarnitine:Alanine; Carnitine:Dodecenoylcarnitine; Tetradecenoyl carnitine:Alanine					
**Late preterm births (34–36 weeks’ gestation)**							
**Model 1** ^**e**^	Infant sex, gestational age, birth weight, plurality, and TPN	0.684	0.683	0.001	7722	—	—
**Model 2**	Model 1 variables + relative fetal-to-adult Hb level	0.687	0.685	0.002	7711	0.00042 (0.0001, 0.0007)	0.10901 (0.04, 0.178)
**Model 3**	Model 2 variables + 17 − OHP + TSH	0.727	0.725	0.002	7588	0.003943 (0.0024, 0.0055)	0.34868 (0.279, 0.4184)
**Model 4** ^**f**^	Model 3 variables + restricted cubic spline terms for the top five ranked analytes/analyte ratios + remaining analytes/analyte ratios until maximum number of parameters was reached (maximum number of parameters: 80)	0.801	0.782	0.019	7086	0.016826 (0.0146, 0.019)	0.69197 (0.6274, 0.7565)
**Top 10 analytes/analyte ratios** ^h^		Dodecanoylcarnitine:Relative fetal-to-adult Hb level; Decanoylcarnitine; Dodecanoylcarnitine; Dodecenoylcarnitine:Relative fetal-to-adult Hb level; Dodecenoylcarnitine:Phenylalanine; Decanoylcarnitine:Relative fetal-to-adult Hb level; Valerylcarnitine:Methylglutarylcarnitine; Malonylcarnitine:17-OHP; Methylglutarylcarnitine; Decanoylcarnitine:Phenylalanine					
**Early preterm births (<34 weeks’ gestation)**							
**Model 1** ^**e**^	Infant sex, gestational age, birth weight, plurality, and TPN	0.653	0.650	0.003	6696	—	—
**Model 2**	Model 1 variables + relative fetal-to-adult Hb level	0.653	0.649	0.004	6695	0.000258 (−0.0002, 0.0007)	0.079332 (0.0166, 0.142)
**Model 3**	Model 2 variables + 17 − OHP + TSH	0.658	0.654	0.004	6678	0.002719 (0.0014, 0.0041)	0.074809 (0.0107, 0.1389)
**Model 4** ^**f**^	Model 3 variables + restricted cubic spline terms for the top five ranked analytes/analyte ratios + remaining analytes/analyte ratios until maximum number of parameters was reached (maximum number of parameters: 102)	0.701	0.667	0.034	6559	0.016748 (0.0136, 0.0199)	0.33787 (0.2746, 0.4011)
**Top 10 analytes/analyte ratios** ^h^		Methylglutarylcarnitine; Valerylcarnitine:Methylglutarylcarnitine; Tyrosine:Valine; Methylmalonylcarnitine:Phenylalanine; Dodecanoylcarnitine:Valine; Methylmalonylcarnitine:Valine; Dodecanoylcarnitine:17-OHP; Octadecenoylcarnitine:Arginine; Dodecanoylcarnitine:Phenylalanine; Hexadecanoylcarnitine:Arginine					

Abbreviations: AIC: Akaike Information Criterion; CI: confidence interval; Hb: hemoglobin; IDI: Integrated Discrimination Improvement; NRI: Net Reclassification Improvement; 17-OHP: 17-hydroxyprogesterone; TPN: total parenteral nutrition; TSH: thyroid stimulating hormone

^a^C-statistic is equivalent to the area under the curve (AUC).

^b^Adjusted c-statistic is based on internal validation results using 200 bootstrap samples.

^c^Integrated Discrimination Improvement (IDI) quantifies the impact of additional variables on the average sensitivity of the preceding nested model (e.g., the IDI shown for Model 2 compares Model 2 with Model 1, etc.).

^d^Net Reclassification Improvement (NRI) quantifies the net increase/decrease in predicted values for the outcome compared to the preceding nested model (e.g., the NRI shown for Model 2 compares Model 2 with Model 1, etc.).

^e^Baseline model containing only clinical variables.

^f^Final fitted model.

^g^C-statistic is unadjusted due to non-convergence of optimism-corrected model.

^h^Rank ordered by absolute value of the regression parameter in Model 4.

#### Late preterm (34–36 weeks)

We observed similar patterns in model performance among infants born at late preterm gestational ages. Compared to the baseline model containing only clinical factors, the addition of the relative fetal-to-adult Hb level (Model 2) did not improve the model fit; however, the optimism-adjusted c-statistic increased when 17-OHP and TSH were added (from 0.685 in Model 2 to 0.725 in Model 3) and again when the additional screening analytes/analyte ratios were added to the final model (adjusted c-statistic increased to 0.782). The IDI also increased significantly in Models 2, 3, and 4 (comparing with Models 1, 2, and 3, respectively); however, to a lesser magnitude than the incremental improvements observed among the term infants (Table 3). Again, in this subgroup, 17-OHP was associated with an increase in the risk of neonatal sepsis; however, there was no association between TSH level and sepsis in the fully-adjusted final model.

#### Early preterm (<34 weeks)

Compared with the other gestational age subgroups, the models did not perform well in early preterm infants. The addition of the relative fetal-to-adult Hb level at birth did not improve the fit of the model beyond the predictive ability of the nested model containing clinical variables only, nor did the addition of other non-mass spectrometry derived newborn screening analytes in Model 3 (TSH or 17-OHP). The c-statistic adjusted for optimism in the final model (Model 4) was 0.667, only minimally different from the baseline model (optimism-adjusted c-statistic 0.65) and of a magnitude indicating poor predictive ability. When we reanalyzed these models including early preterm infants who had received a transfusion, the adjusted c-statistic showed little change across the models (0.74 in Model 1 and 0.75 in Model 4).

### Heat map analysis

Supplementary Figs S1 through S6 present correlational heat maps for infants with and without a diagnosis of neonatal sepsis. Among term infants, with or without sepsis, the strongest positive correlation was between carnitine and acetylcarnitine (ρ = 0.8, Supplementary Figs S1 and S2), while the strongest negative correlation was between methylmalonylcarnitine and relative fetal-to-adult Hb level (ρ = −0.3, Supplementary Figs S1 and S2). The strongest positive and negative correlations among non-septic infants born at late preterm gestation were also among acyl-carnitines and of similar magnitude (Supplementary Fig. S3); however, among septic infants born at late preterm gestation, carnitine and acetylcarnitine (ρ = 0.9) and leucine and TSH (ρ = −0.3) were the strongest positive and negative correlations (Supplementary Fig. S4). Among very preterm infants, the majority of correlations between pairs of analytes were negative, irrespective of sepsis (Supplementary Figs S5 and S6).

## Discussion

Reducing the burden and harms associated with neonatal sepsis is an international priority warranting further exploration of the potential for early detection of this condition^28^. In this large, population-based study we assessed whether analyte values from newborn screening samples taken close to the time of birth demonstrated the potential to identify infants at higher likelihood of developing sepsis during the neonatal period. Our results indicate that the newborn screening profiles of infants with neonatal sepsis are different from those of non-septic infants. In particular, a combination of baseline clinical variables and newborn screening analytes performed well in identifying neonatal sepsis among late preterm and term infants. In these two gestational age groups, clinical variables alone or in combination with hemoglobin values were not strongly predictive of developing neonatal sepsis. However, the model fit improved considerably after adding markers of thyroid and adrenal dysfunction, along with acyl-carnitines, enzyme makers, and amino acids, with adjusted c-statistic values indicating a strong model for identifying cases of neonatal sepsis. However, among infants born at early preterm gestational ages (i.e., <34 weeks), clinical variables alone were markedly less predictive of neonatal sepsis and the addition of newborn screening analytes minimally altered the predictive ability of the model. These results provide insights into the pathophysiology of neonatal sepsis and may provide guidance for the identification of early biomarkers.

Different analyte patterns associated with neonatal sepsis in infants could reflect differences in underlying physiology that may predispose infants towards the condition. For example,various studies have suggested that elevated fetal hemoglobin levels may predispose infants to adverse health outcomes, including neonatal sepsis^29–31^. Tighter oxygen binding capacity and thus inferior oxygen delivery to tissues may play a role in susceptibility to sepsis. However, in our models, the addition of fetal hemoglobin to baseline clinical characteristics had no impact on the model’s ability to identify infants with neonatal sepsis in any of the gestational age groups. We also hypothesized *a priori* that measures of thyroid and adrenal function (TSH and 17-OHP, respectively) could be associated with neonatal sepsis. A low level of cortisol is a marker of inadequate adrenal response to illness^32,33^, and links have previously been identified between levels of TSH and neonatal sepsis^34^. We found the addition of markers of thyroid and adrenal function did improve the model fit, but not as substantially as the addition of the acyl-carnitines and amino acids.

Alternatively, different analyte patterns associated with neonatal sepsis in infants could reflect the stress response to sepsis. Our analyses demonstrate that in late preterm and term infants, acyl-carnitines, enzyme markers, and amino acids are the newborn screening analytes most strongly associated with neonatal sepsis. Associations between acyl-carnitines and sepsis have been previously documented, with heightened analyte levels occurring among septic infants^35,36^ and positive correlations being reported between certain acyl-carnitine levels and 28-day mortality^37^. High acyl-carnitine levels have also been reported in other cases of injury^35^ and catabolic stress, which is likely the mechanism of their association with neonatal sepsis^38–40^. In cases of stress, fatty acid oxidation is disrupted, resulting in higher levels of circulating acyl-carnitines^36^. Abnormal carnitine metabolism can then contribute to the development of septic shock and multi-system organ damage^36^. Our hypothesis that catabolic stress mediated the associations we observed also explains the model performing less well among early preterm infants, many of whom experience catabolic stress even in the absence of sepsis^41^. However, the changes in acyl-carnitines and amino acids we observed may also reflect differences in metabolic processes that could predispose infants towards neonatal sepsis. In several of the conditions screened for with newborn screening, such as medium-chain acyl-CoA dehydrogenase deficiency, an infectious stressor can result in metabolic decompensation^42^. The subtle alterations we observed may reflect milder changes in the same pathways that are altered in these conditions.

A strength of our study was the size of the cohort — after exclusions, we were able to analyze data from nearly 800,000 newborns, 4,800 of whom received a diagnosis of sepsis during the neonatal period. This large, population-based cohort provided substantial statistical power to perform the complex analyses needed to develop our models. Nevertheless, our study also had several important limitations. The exact timing of onset of clinical signs of neonatal sepsis was not documented in our data, and could have occurred prior to blood spot collection, which typically occurs between 24 and 72 hours after birth. In the hospitalization database, we were also unable to reliably distinguish between early- and late-onset sepsis. Since early- and late-onset disease may differ in their clinical presentation and etiology^3^, it is possible that newborn screening analytes could also display distinct patterns according to the timing of onset. In addition, we did not capture diagnoses of late-onset sepsis after 28 days of age. Finally, the use of diagnostic codes in administrative databases to identify cases of neonatal sepsis is expected to generate some measurement error due to low sensitivity of the codes^28^. Although it is likely that many of the infants who received an ICD-10-CA code for sepsis (P36) in the hospitalization database had a confirmed diagnosis of sepsis based on blood culture, we could not distinguish these confirmed diagnoses from infants classified on the basis of possible sepsis. False-negative classification of neonatal sepsis owing to low sensitivity would be expected to dilute the strength of our findings. The low prevalence of neonatal sepsis could also result in more false-positive results despite the high specificity; however, we expect any false-positive classification of sepsis is likely identifying other serious neonatal illness also causing catabolic stress. To our knowledge, the diagnostic codes for sepsis have not been validated against medical record data, and this would be informative for future studies^43^.

Our findings have value in identifying future directions for the early detection of neonatal sepsis. Future studies should examine whether measurement of markers of catabolic stress soon after birth can help identify neonatal sepsis in infants who would otherwise not be considered at risk for the condition. Our findings complement those recently published by Sarafidis and colleagues^44^, whose study found substantial differences in the metabolic profiles of urine samples from septic and non-septic infants. As with our study, they also noted significantly heightened levels of several amino acids among late-onset neonatal sepsis cases, which the authors hypothesized may suggest disturbances in protein metabolism^44^.

## Conclusion

In this study, we sought to assess whether there was any association between newborn screening analyte profiles and neonatal sepsis. Our models suggest potential utility in using a combination of clinical variables and newborn screening analytes to identify neonatal sepsis among infants born at term or late preterm gestational ages, among whom illness may not otherwise be suspected. Indications of catabolic stress in newborn screening analyte values can serve to prompt clinicians to further explore the possibility of sub-clinical or impending illness, potentially resulting in earlier diagnosis and treatment, and ultimately improved health outcomes.

## Methods

### Ethical approval and data access

This study was conducted at the Institute for Clinical Evaluative Sciences (ICES), a non-profit research organization with Prescribed Entity status under provincial privacy legislation. ICES acts as a secure repository for Ontario’s newborn screening database and provincial health administrative databases, with the ability to deterministically link individual patient health information across databases using unique encoded identifiers to protect privacy and confidentiality. This study received approval from the institutional review board at Sunnybrook Health Sciences Centre, the ICES Privacy Office, the Ottawa Health Science Network Research Ethics Board, and the Children’s Hospital of Eastern Ontario Research Ethics Board. All procedures were performed in accordance with these institutions’ relevant guidelines and regulations. Given the Prescribed Entity status of ICES under Ontario’s privacy legislation, express consent was not required for the use of the anonymized health administrative data used in this study. The dataset from this study is held securely in coded form at ICES. While data sharing agreements prohibit ICES from making the dataset publicly available, access may be granted to those who meet pre-specified criteria for confidential access, available at www.ices.on.ca/DAS. The full dataset creation plan is available from the authors upon request.

### Study design, population and data sources

We conducted a population-based retrospective cohort study of all live births in the province of Ontario between January 1, 2010 and December 30^th^, 2015 with completed newborn screening. Infants were identified using electronic screening records from Newborn Screening Ontario (NSO), a province-wide program that screens all infants for rare conditions using a panel of screening analytes obtained via heel prick blood spot, typically between 24 and 72 hours following birth. Each infant’s electronic screening record contained information on sex, gestational age (completed weeks), mode of feeding (i.e., breast, formula, total parenteral nutrition), timing of blood spot collection, transfusion of blood or blood products, and specific analyte values.

We excluded infants who screened positive for one or more disorders on the newborn screening panel, those with unsatisfactory sample quality, and those who were transfused prior to blood spot collection, as donor blood interferes with hemoglobin and galactosemia-related analytes. We additionally excluded those without continuous eligibility for provincial health care benefits between birth and the end of the neonatal period (i.e., up to 28 days of age). To ascertain the study outcome, we linked the study cohort to hospitalization records from the Canadian Institute for Health Information’s Discharge Abstract Database (DAD) using unique encrypted identifiers at ICES. For each admission of an infant to any Ontario hospital (including the birth hospitalization, any neonatal intensive care unit transfers/admissions or any other hospital admission), the DAD contains information on up to 25 medical diagnoses (coded using the Canadian implementation of the International Classification of Diseases, 10th Revision [ICD-10-CA]^45^), medical interventions received, length of hospital stay and other data elements. Only hospitalizations with a date of admission within the neonatal period were included.

### Variable measurement

All newborn screening samples in Ontario are analyzed in one central laboratory using standardized biochemical assays and reported in specific measurement units for each analyte. The 49 individual analytes used in our study (Table 4) included acyl-carnitines (n = 31), amino acids (n = 12), relative fetal-to-adult hemoglobin level (n = 1), endocrine markers (n = 2), and enzymes (n = 3). To minimize the impact of extreme analyte values in this screen-negative study population, we replaced the outliers (i.e., those below the 0.001^st^ percentile or above the 99.999^th^ percentile) with the analyte value at those percentile levels. We computed analyte ratio combinations (n = 1,176) to capture the relative balance/imbalance between individual analytes after replacing any analyte value of zero with the next lowest positive value in the study population in order to have a mathematically valid denominator. We standardized all analytes to the standard normal distribution to render a unit change in each analyte comparable regardless of different measurement scales. This standardization was conducted by calendar week of birth of the screened infant to minimize variation due to any external factors, including changes in assays, seasonal effects, or other non-biological factors over the study period.

**Table 4:** Newborn screening analytes used in model development.

Acyl-carnitines (n = 31)	C0 (carnitine)	C16 (hexadecanoylcarnitine)
	C2 (acetylcarnitine)	C18 (octadecanoylcarnitine)
	C3 (propionylcarnitine)	C18:1 (octadecenoylcarnitine)
	C4 (butyrylcarnitine)	C18:2 (octadecadienylcarnitine)
	C5 (valerylcarnitine)	C4OH (hydroxybutyrylcarnitine)
	C5:1 (Tiglylcarnitine)	C5DC (glutarylcarnitine)
	C6 (hexanoylcarnitine)	C5OH (hydroxyvalerylcarnitine)
	C8 (octanoylcarnitine)	C6DC (methylglutarylcarnitine)
	C8:1 (octenoylcarnitine)	C14:OH (3-hydroxytetradecanoylcarnitine)
	C10 (decanoylcarnitine)	C16:OH (hydroxyhexadecanoylcarnitine)
	C10:1 (decenoylcarnitine)	C16:1OH (hydroxyhexadecenoylcarnitine)
	C12 (dodecanoylcarnitine)	C18OH (3-hydroxystearoylcarnitine)
	C12:1 (dodecenoylcarnitine)	C18:1OH (hydroxyoctadecenoylcarnitine)
	C14 (tetradecanoylcarnitine)	C3DC (malonylcarnitine)
	C14:1 (tetradecenoyl carnitine)	C4DC (methylmalonylcarnitine)
	C14:2 (tetradecadienylcarnitine)	
Amino acids and related markers (n = 12)	Arginine	Tyrosine
	Phenylalanine	Glycine
	Alanine	Argininosuccinate
	Leucine	Methionine
	Ornithine	Valine
	Citrulline	Succinylacetone
Relative fetal-to-adult hemoglobin (Hb) level (n = 1)	Fetal hemoglobin (HbF + HbF1)/(Fetal hemoglobin (HbF + HbF1) + Adult hemoglobin (HbA))	
Endocrine markers (n = 2)	17-hydroxyprogesterone (17-OHP)	
	Thyroid stimulating hormone (TSH)	
Enzyme markers (n = 3)	Biotinidase (BIOT)	
	Galactose-1-Phosphate Uridyltransferase (GALT)	
	Immunotripsinogen (IRT)	

We defined neonatal sepsis cases as those infants with a diagnostic code for newborn sepsis (ICD-10-CA code: P36) recorded during any hospitalization initiated within the neonatal period (i.e., prior to 28 days of age), but we were unable to determine the exact time of onset or distinguish between early- or late-onset cases. A Canadian validation study of diagnostic codes for neonatal sepsis using administrative health data found sensitivity and specificity values of 38.4% and 99.7%, respectively, for the P36 code when validated against a clinical perinatal database^28^.

### Statistical analyses

We described baseline characteristics of the study population using frequency distributions and descriptive statistics, and calculated crude rates of neonatal sepsis per 1,000 screened infants. As gestational age is strongly correlated with both incidence of neonatal sepsis and with newborn screening analyte values^46–49^, we made an *a priori* decision to stratify all remaining analyses into subgroups defined by gestational age: <34 weeks (early preterm), 34–36 weeks (late preterm), and ≥37 weeks (term). To improve the statistical efficiency of predictive modelling within the large group of term infants, we subset the data by randomly selecting 10 non-septic infants for every infant diagnosed with sepsis.

To avoid over-fitting and conserve degrees of freedom in our gestational age-specific models, *a priori*, we determined the maximum number of model parameters that could be accommodated in the analysis for each gestational age group, based on a requirement of 10 sepsis cases per parameter. We then used several steps to reduce the number of analytes and analyte ratios prior to modeling. First, we computed crude Spearman’s rank correlation coefficients between sepsis and each individual standardized analyte value and standardized analyte ratio. We then computed partial Spearman’s correlations between sepsis and all individual standardized analytes, as well as the top 100 ranked standardized analyte ratios based on crude Spearman’s correlation (top 50 ranked standardized analyte ratios for the late preterm birth group owing to the low number of sepsis cases in that gestational age stratum), adjusted for infant sex, gestational age, birth weight, plurality, and mode of feeding. We then prioritized variables for inclusion in multivariable modelling in order of magnitude of partial Spearman’s correlation, and the constraint requiring at least 10 sepsis cases for every parameter included in the model. Variables modelled categorically, or using restricted cubic splines required additional degrees of freedom and thus represented more than one regression parameter.

The models were developed using multivariable logistic regression with sepsis as the binary dependent variable. We chose models based on both pathophysiological parameters as well as mechanism of measurement. We began with a baseline model (Model 1) containing only the following clinical factors: infant sex (male vs. female), continuous gestational age (weeks), continuous birth weight (grams), plurality (singleton vs. twin or higher-order multiple), and mode of feeding (total parenteral nutrition vs. other). To this baseline clinical model, we then added the relative fetal-to-adult hemoglobin (Hb) level (Model 2), which represents a marker of oxygen delivery to tissues and is measured using high performance liquid chromatography. Next, markers of endocrine function and analytes measurable using non-mass spectrometry methods were added in Model 3 (thyroid stimulating hormone [TSH] and 17-hydroxyprogesterone [17-OHP]). In the final model (Model 4), we added the remaining analytes/analyte ratios (acyl-carnitines, enzyme markers, and amino acids) according to rank order based on Spearmans’s correlation until the maximum number of parameters was reached. We used restricted cubic spline terms to model the top five ranked analytes/analyte ratios to capture non-linear associations. The decision of how many analytes to model using splines was made *post-hoc* by inspection of the ranked partial Spearman’s correlations, and using splines to model a small number of the top analytes if there was a sharp drop-off in correlation after the first few ranked analytes. In a sensitivity analysis of the early preterm subgroup, we re-ran the models while including infants with a documented transfusion of blood or blood products, and adjusted for transfusion in the first nested model containing only clinical variables (Model 1) as well as all subsequent models.

We assessed model discrimination using the area under the receiver operating characteristic curve (AUC) and used the Integrated Discrimination Improvement (IDI)^50^ to quantify the impact of additional groups of analytes on the average sensitivity of the preceding nested model. We also computed the Net Reclassification Improvement (NRI) to quantify the net increase/decrease in predicted values for the outcome compared to the preceding nested model^50^. Since we were unable to partition our dataset into random subsamples for validation purposes owing to the low prevalence of sepsis, we internally validated our model performance using a bootstrap approach as described by Harrell *et al*.^51^. We drew repeated samples with replacement to produce 200 bootstrap samples, each the same size as the original dataset. The average difference in c-statistic values between the original model and each bootstrap sample was used to calculate an optimism-adjusted c-statistic^51^ and we additionally report the Akaike Information Criterion (AIC) for each model, which is a metric that rewards model goodness of fit, but includes a penalty against a large number of parameters to reduce overfitting. Lower AIC values are indicative of improved model fit, relative to preceding nested models. We generated correlation heat maps to visualize the relationships between analytes and differences in correlation patterns between infants with and without neonatal sepsis. Analyses were conducted with SAS version 9.4 (SAS Institute, Inc., Cary, North Carolina) and with R/R Studio using the RMS and HMISC packages.

## Electronic supplementary material

Supplementary Information
