# Supplementary material for: Using newborn screening analytes to identify cases of neonatal sepsis
Source: Sci Rep. 2017 Dec 21;7:18020. doi: 10.1038/s41598-017-18371-1 (PMC5740154; doi:10.1038/s41598-017-18371-1)

**Full title:** Using newborn screening analytes to identify cases of neonatal sepsis

**Authors:** Deshayne B. Fell[1,2,3], Steven Hawken[1,3,4], Coralie A. Wong[3], Lindsay A. Wilson[4], Malia S.Q. Murphy[4], Pranesh Chakraborty[5,6], Thierry Lacaze-Masmonteil[7], Beth K. Potter,[1,3] Kumanan Wilson[3,4,8] *

**Authors' institutional affiliations:**

1. School of Epidemiology and Public Health, University of Ottawa, Ottawa Ontario, Canada
2. Children's Hospital of Eastern Ontario Research Institute, Ottawa Ontario, Canada
3. Institute for Clinical Evaluative Sciences (ICES), University of Ottawa, Ottawa Ontario, Canada
4. Clinical Epidemiology Program, Ottawa Hospital Research Institute, Ottawa Ontario, Canada
5. Department of Pediatrics, University of Ottawa, Ottawa Ontario, Canada
6. Newborn Screening Ontario (NSO), Children's Hospital of Eastern Ontario, Ottawa Ontario, Canada
7. Department of Pediatrics, University of Calgary, Calgary Alberta, Canada
8. Department of Medicine, University of Ottawa, Ottawa Ontario, Canada

***Corresponding author:** Dr. Kumanan Wilson; Institute for Clinical Evaluative Sciences, 1053 Carling Avenue, Administrative Services Building, Room 1009, Box 684; Ottawa, Ontario, Canada K1Y 4E9; Tel: 613-798-5555 ext. 17921; Fax: 613-761-5492; E-mail: kwilson@ohri.ca

**Supplementary Figure S1. Correlation heat map of analytes for infants born at term gestation with no diagnosis of neonatal sepsis.** Darker green areas indicate higher levels of positive correlation between analytes, and darker red areas indicate higher levels of negative correlation between analytes. Full names of all analytes are shown in Table 4 of the manuscript.

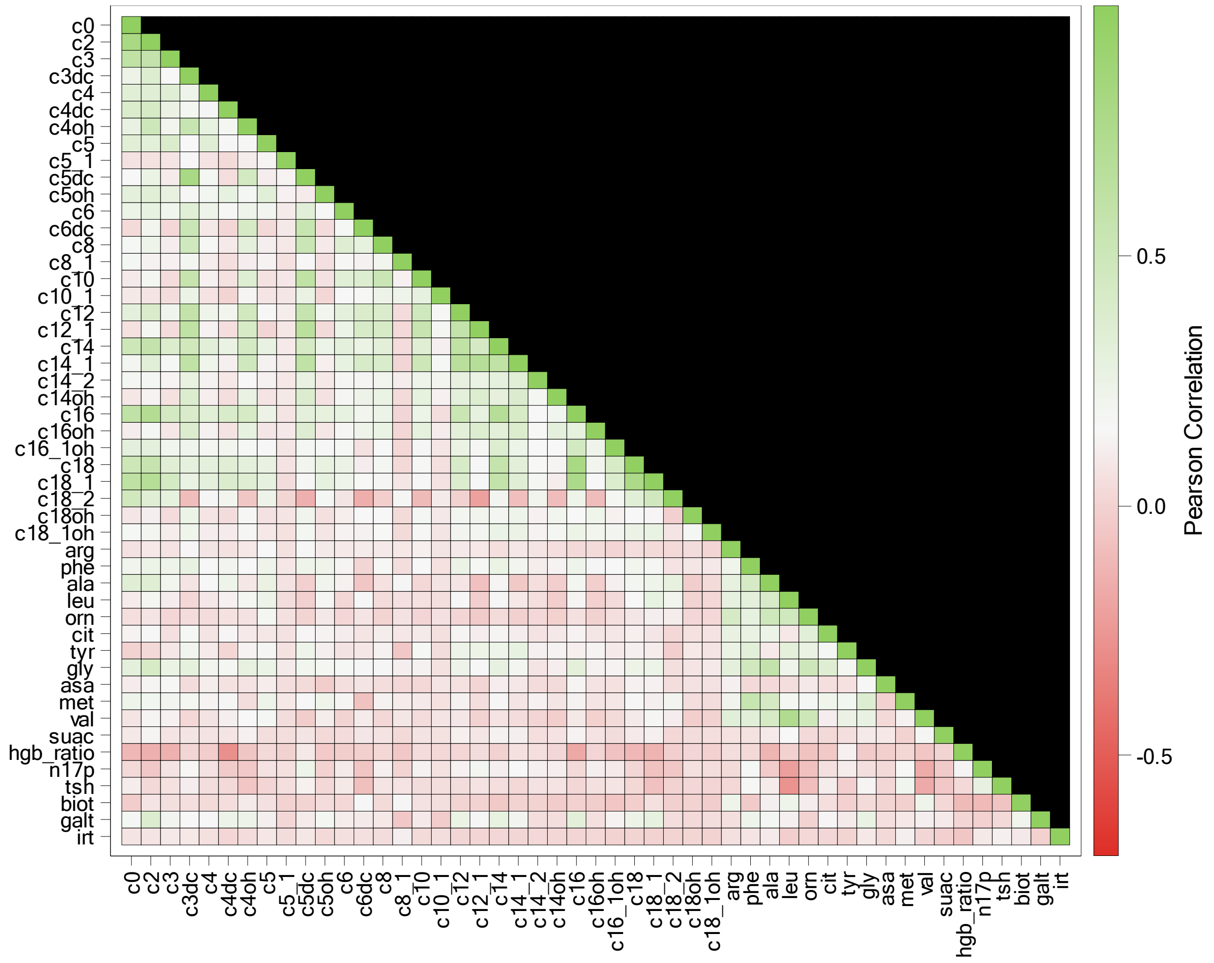

**Supplementary Figure S2. Correlation heat map of analytes for infants born at term gestation with a diagnosis of neonatal sepsis.** Darker green areas indicate higher levels of positive correlation between analytes, and darker red areas indicate higher levels of negative correlation between analytes. Full names of all analytes are shown in Table 4 of the manuscript.

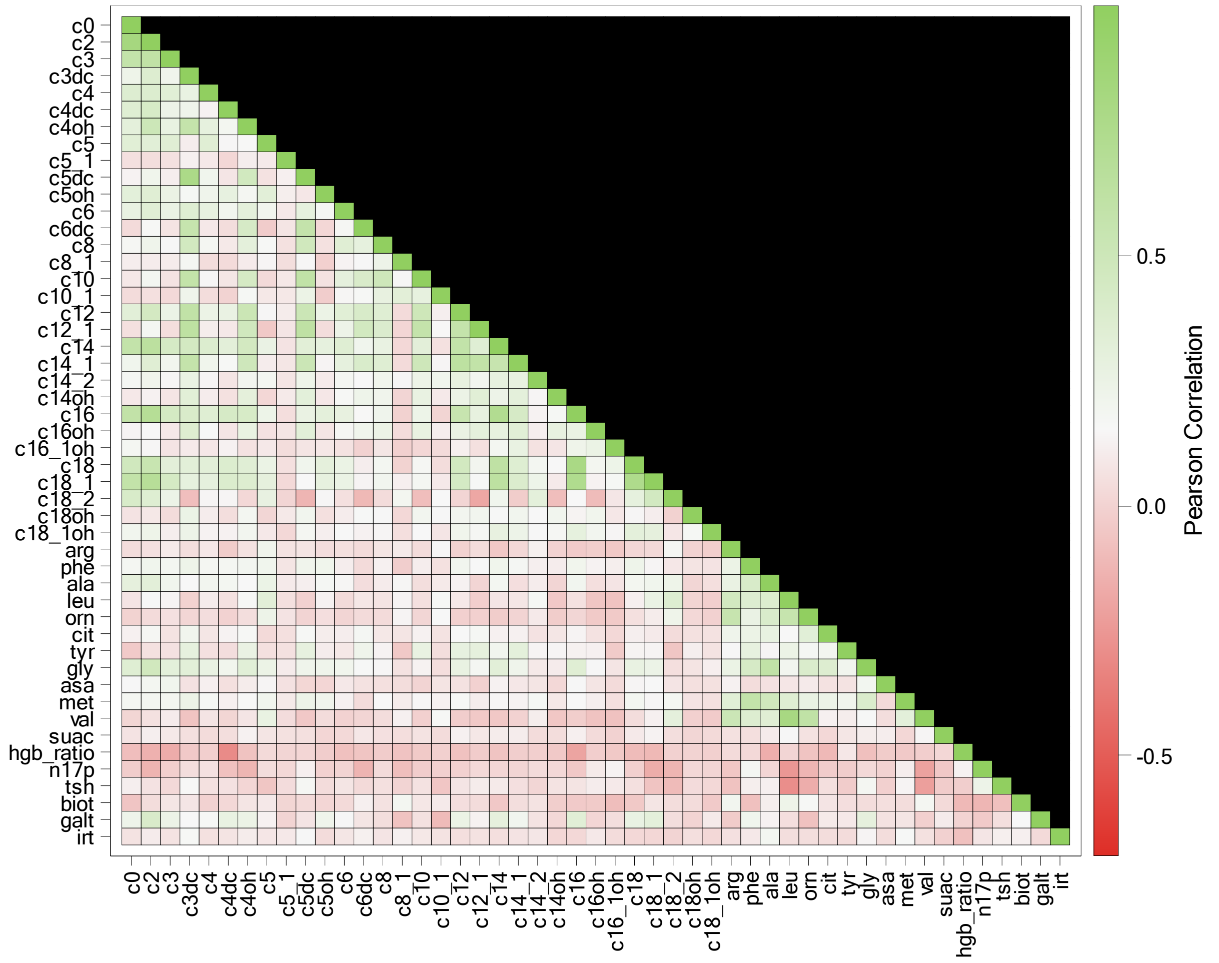

**Supplementary Figure S3. Correlation heat map of metabolites for infants born at late preterm gestation (i.e., 34-36 weeks) with no diagnosis of neonatal sepsis.** Darker green areas indicate higher levels of positive correlation between analytes, and darker red areas indicate higher levels of negative correlation between analytes. Full names of all analytes are shown in Table 4 of the manuscript.

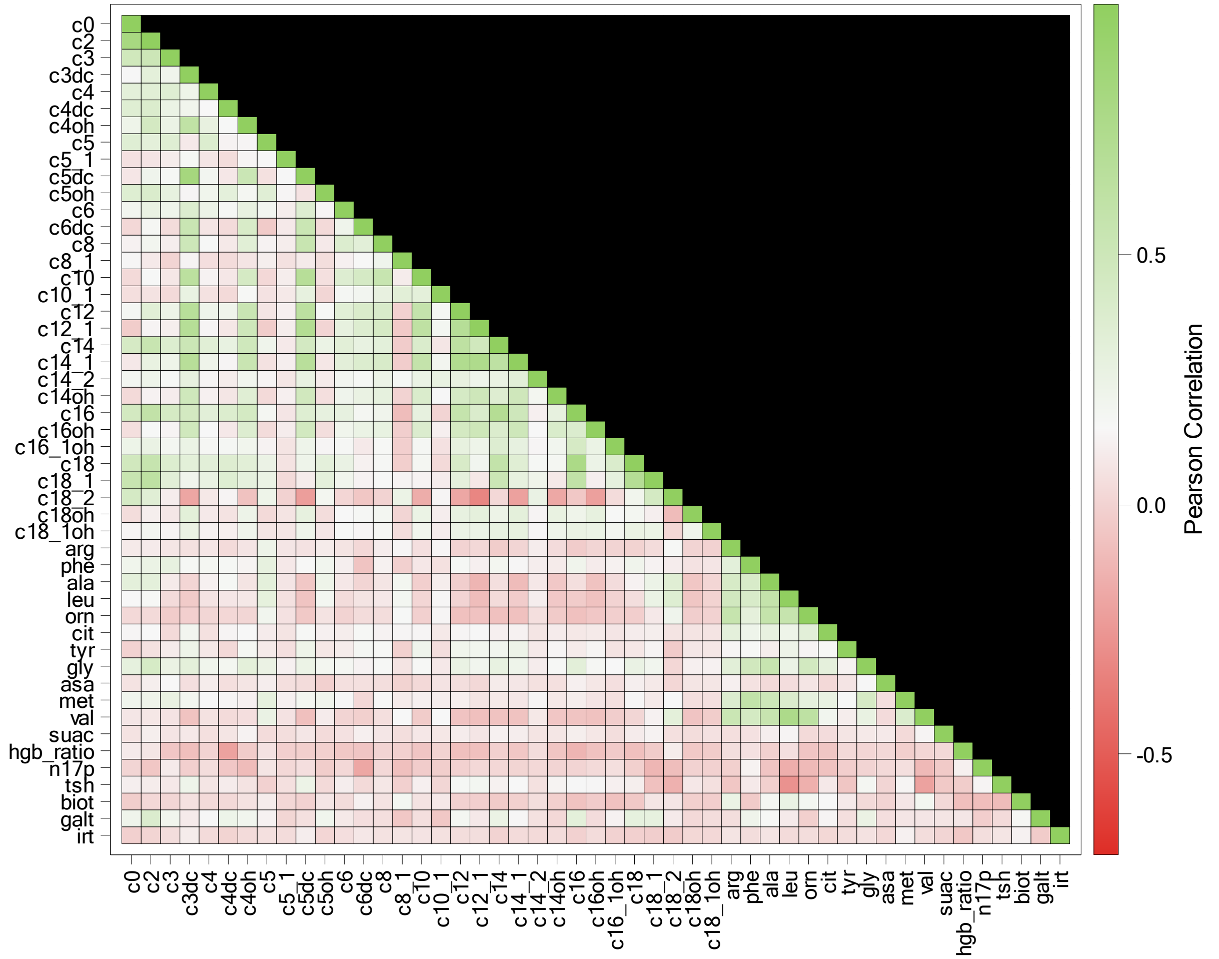

**Supplementary Figure S4. Correlation heat map of analytes for infants born at late preterm gestation (i.e., 34-36 weeks) with a diagnosis of neonatal sepsis.** Darker green areas indicate higher levels of positive correlation between analytes, and darker red areas indicate higher levels of negative correlation between analytes. Full names of all analytes are shown in Table 4 of the manuscript.

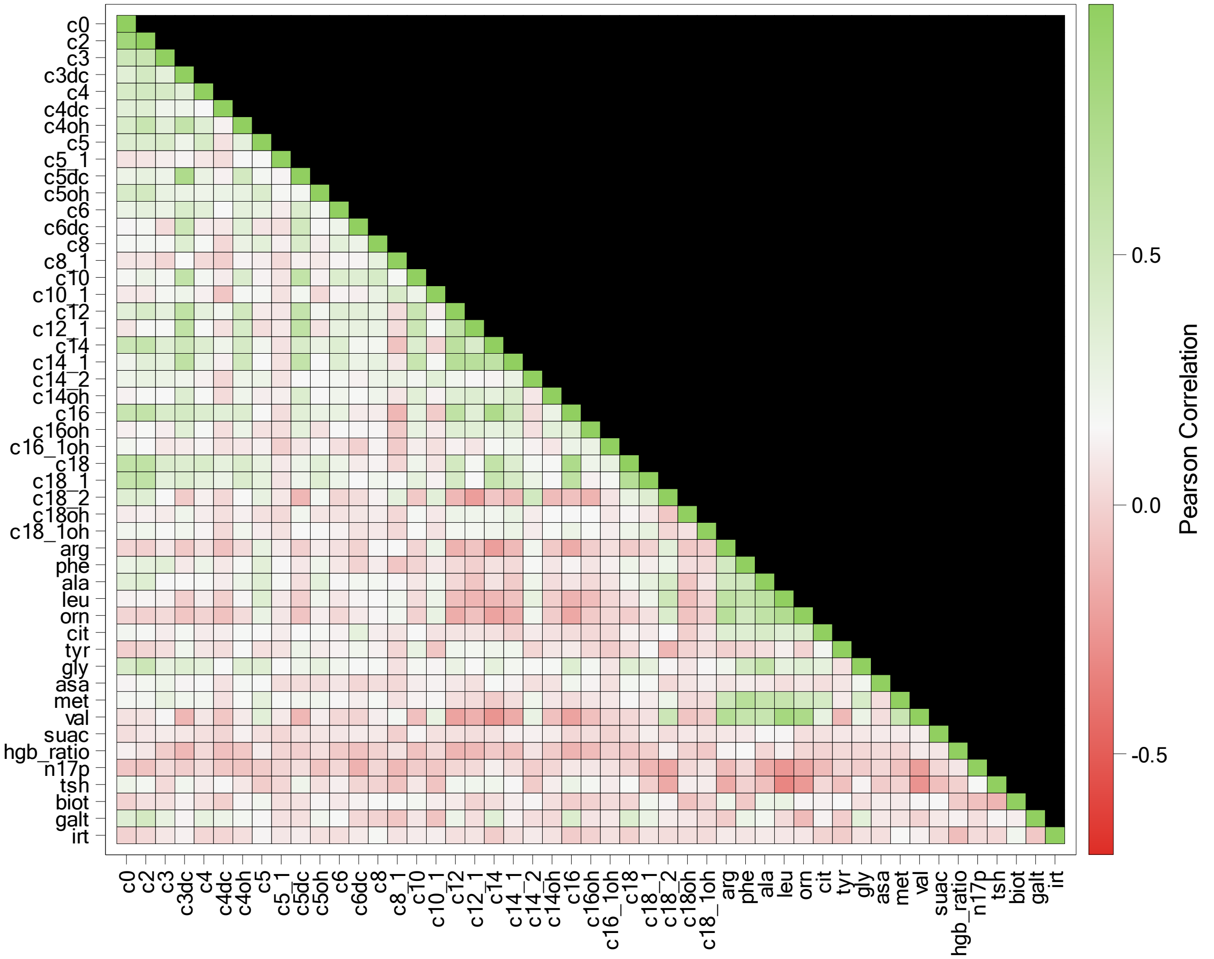

**Supplementary Figure S5. Correlation heat map of analytes for infants born at early preterm gestation (i.e., <34 weeks) with no diagnosis of neonatal sepsis.** Darker green areas indicate higher levels of positive correlation between analytes, and darker red areas indicate higher levels of negative correlation between analytes. Full names of all analytes are shown in Table 4 of the manuscript.

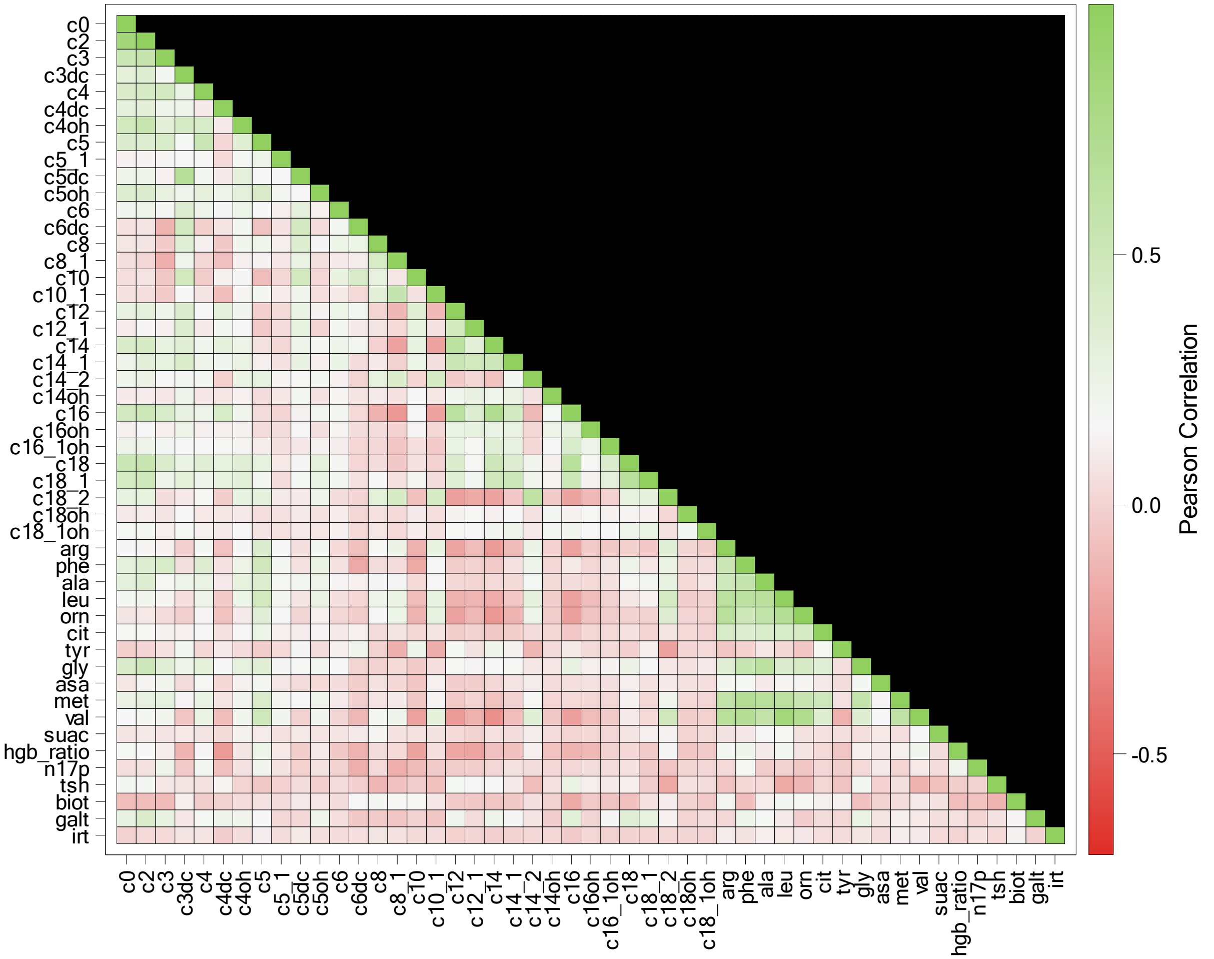

**Supplementary Figure S6. Correlation heat map of analytes for infants born at early preterm gestation (i.e., <34 weeks) with a diagnosis of neonatal sepsis.** Darker green areas indicate higher levels of positive correlation between analytes, and darker red areas indicate higher levels of negative correlation between analytes. Full names of all analytes are shown in Table 4 of the manuscript.

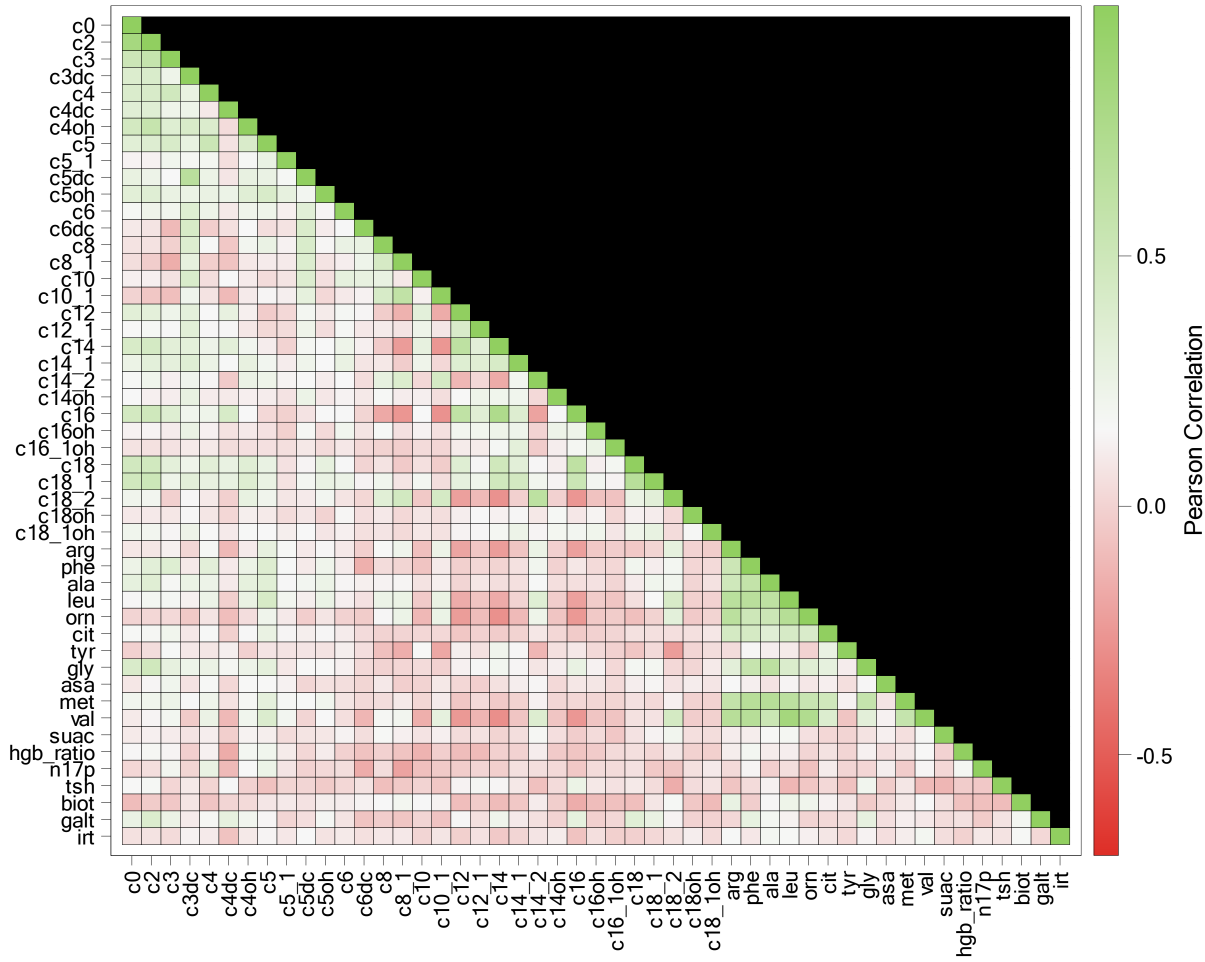

Supplement: Supplementary file 1 — Supplementary Information [file 41598_2017_18371_MOESM1_ESM.pdf]
